# Development of a Deep Learning Model for Dynamic Forecasting of Blood Glucose Level for Type 2 Diabetes Mellitus: Secondary Analysis of a Randomized Controlled Trial

**DOI:** 10.2196/14452

**Published:** 2019-11-01

**Authors:** Syed Hasib Akhter Faruqui, Yan Du, Rajitha Meka, Adel Alaeddini, Chengdong Li, Sara Shirinkam, Jing Wang

**Affiliations:** 1 Department of Mechanical Engineering University of Texas at San Antonio San Antonio, TX United States; 2 Center on Smart and Connected Health Technologies University of Texas Health Science Center at San Antonio San Antonio, TX United States; 3 Department of Mathematics and Statistics University of the Incarnate Word San Antonio, TX United States

**Keywords:** type 2 diabetes, long short-term memory (LSTM)-based recurrent neural networks (RNNs), glucose level prediction, mobile health lifestyle data

## Abstract

**Background:**

Type 2 diabetes mellitus (T2DM) is a major public health burden. Self-management of diabetes including maintaining a healthy lifestyle is essential for glycemic control and to prevent diabetes complications. Mobile-based health data can play an important role in the forecasting of blood glucose levels for lifestyle management and control of T2DM.

**Objective:**

The objective of this work was to dynamically forecast daily glucose levels in patients with T2DM based on their daily mobile health lifestyle data including diet, physical activity, weight, and glucose level from the day before.

**Methods:**

We used data from 10 T2DM patients who were overweight or obese in a behavioral lifestyle intervention using mobile tools for daily monitoring of diet, physical activity, weight, and blood glucose over 6 months. We developed a deep learning model based on long short-term memory–based recurrent neural networks to forecast the next-day glucose levels in individual patients. The neural network used several layers of computational nodes to model how mobile health data (food intake including consumed calories, fat, and carbohydrates; exercise; and weight) were progressing from one day to another from noisy data.

**Results:**

The model was validated based on a data set of 10 patients who had been monitored daily for over 6 months. The proposed deep learning model demonstrated considerable accuracy in predicting the next day glucose level based on Clark Error Grid and ±10% range of the actual values.

**Conclusions:**

Using machine learning methodologies may leverage mobile health lifestyle data to develop effective individualized prediction plans for T2DM management. However, predicting future glucose levels is challenging as glucose level is determined by multiple factors. Future study with more rigorous study design is warranted to better predict future glucose levels for T2DM management.

## Introduction

Diabetes mellitus is a serious health condition resulting from defects of insulin secretion and/or insulin action [1]. Patients with type 2 diabetes mellitus (T2DM) need to maintain strict glycemic control to avoid the risk of hypoglycemia, hyperglycemia, and consequential complications [2]. T2DM, characterized by the combination of insufficient insulin secretion and insulin resistance, accounts for approximately 90% to 95% of all diabetes cases [3]. It has become a major public health concern as it is burdensome for individuals, health systems, and society [4]. Self-management of diet, physical activity, weight, and medication and self-monitoring of blood glucose are essential for glycemic control [5,6]. However, it is very challenging to adhere to this self-management regimen [7].

Emerging evidence has demonstrated that mobile technologies can promote a healthy lifestyle and medication adherence and improve diabetes outcomes [8,9]. The underlying mechanisms might include the frequent reminder for blood glucose monitoring [10], self-awareness and control of diabetes [11,12], or behavior adjustment based on tracked behaviors [13]. For instance, Padhye et al [11] reported that in patients with T2DM, smartphone users are more likely to adhere to self-monitoring of diet, physical activity, blood glucose, and body weight when compared with paper diary users. Many studies have evidenced that compliance with self-monitoring of diet and physical activity can lead to weight loss [14] and hemoglobin A_1c_ (HbA_1c_) improvement through behavior adjustment [8,15]. However, digital diabetes care has shown only modest HbA_1c_ improvement in multiple studies [16]. Despite the modest effects of digital self-monitoring on HbA_1c_, recorded lifestyle data may shed light on improving glycemic control through predicting blood glucose level.

There are several algorithms, such as Biostator (Miles-Ames), for automated insulin delivery in order to improve blood glucose control [2,17]. Meanwhile, with an ever-growing amount of data, several machine learning techniques are being developed to understand patterns and develop models that predict the health conditions of patients [18]. For instance, Plis et al [19] described a generic physiological model of blood glucose dynamics to extract informative features to train a support vector regression model on patient-specific data [20-22]. Model predictive control is also used to avoid long delays and open-loop characteristics of the control algorithms [23]. As the relation between input features and glucose levels is nonlinear, dynamic, interactive, and patient-specific, nonlinear regression models are used to build the predictive models [24]. Specifically, neural networks have increasingly been used to model glucose levels using multilayer perceptrons [25,26], time series convolution neural networks, recurrent neural networks [27], convolutional recurrent neural networks [28], and deep convolutional neural networks [29]. Quchani et al [30] compared multilayer perceptron neural networks with Elman recurrent neural networks for predicting the glucose level in patients with type 1 diabetes mellitus (T1DM) to show improvement in the accuracy of the model using recurrent neural networks.

However, to what extent self-monitoring data on health behaviors and weight can help predict blood glucose level in T2DM patients has rarely been studied. Available literature exploring glucose prediction in T2DM mainly focuses on glucose responses to nutrition [31]. However, glucose level is determined by a variety of factors [31-33], and a prediction model incorporating multiple lifestyle factors in a real-world setting is needed. In addition, most of the existing machine learning models predict glucose level for a very short interval (ie, a few minutes [34]), which makes it difficult to plan for effective control strategies. By using long short-term memory (LSTM)-based recurrent neural networks (RNNs), this study aimed to dynamically forecast the next-day glucose levels in individuals with T2DM based on their daily mobile health lifestyle data on diet, physical activity, weight, and previous glucose levels. The study also developed a transfer learning strategy to cope with data scarcity and improve prediction accuracy for individual patients. Additionally, the study used the advanced design of experiments to optimize the hyperparameters of the LSTM-based RNN model.

## Methods

**Figure 1:**
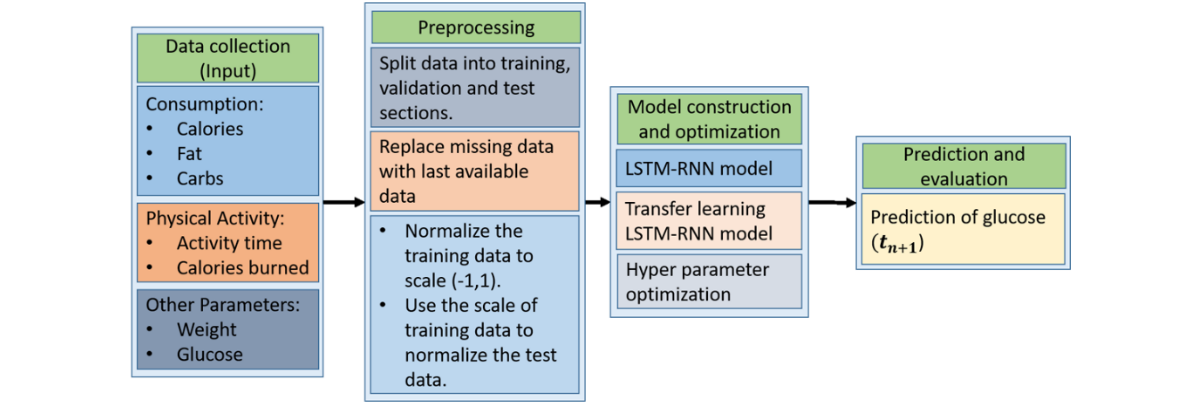
General scheme of the proposed method of predicting blood glucose level. LSTM-RNN: long short-term memory recurrent neural networks.

Forecasting the glucose level of a T2DM patient is critical in planning for future medication and food habits. This study was a secondary analysis of data collected by a randomized controlled trial (RCT) consisting of several steps including data collection, data preprocessing, model construction and optimization, and prediction and evaluation (Figure 1).

### Data Collection

Our study used data collected from a smartphone group based in a pilot randomized trial [11]. The details of the original pilot RCT were published elsewhere [9]. In the randomized trial, overweight/obese adults (BMI >25 kg/m^2^) who were literate in English and diagnosed with T2DM for at least 6 months were eligible to participate. A total of 26 participants aged between 21 and 75 years were enrolled and randomly assigned to a smartphone group (n=11), paper diary group (n=9), and control group (n=6). Participants in the smartphone group received a standard behavioral lifestyle education. The Lose It! (FitNow, Inc) smartphone app was used in this group to self-monitor physical activity, diet, and weight. Blood glucose levels were collected using MyGlucoHealth, a Bluetooth-enabled glucometer (Entra Health Systems) and the DiabetesConnect app (PHRQL, Inc). Informed consent was obtained from each participant, and the study was approved by the Committee for the Protection of Human Subjects at the University of Texas Health Science Center at Houston. One participant in the smartphone group withdrew and did not complete the study.

The data in the smartphone group included an abundance of dynamically monitored lifestyle and health information that has not been fully explored and deserves further mining and analysis to generate study results and provide suggestions and directions for future studies and practices to improve health outcomes. The data collected from the clinical trial was a good fit for our study objective of predicting glucose levels. The 10 participants who were in the smartphone group and recorded at least 150 days of self-monitored data were included in this study. The data for each participant include daily diet information, where collected food intake data (breakfast, lunch, dinner, snacks) is discretized into calories, macronutrient content (carbohydrates and fat), physical activity (where exercise time is translated into calories burned from standard food nutrient charts), weight, and glucose levels. A descriptive summary of the data is presented in Table 1. From the table, it can be seen that patients 1, 2, 4, and 9 have the highest number of missing values in terms of self-reported blood glucose values.

**Figure 2:**
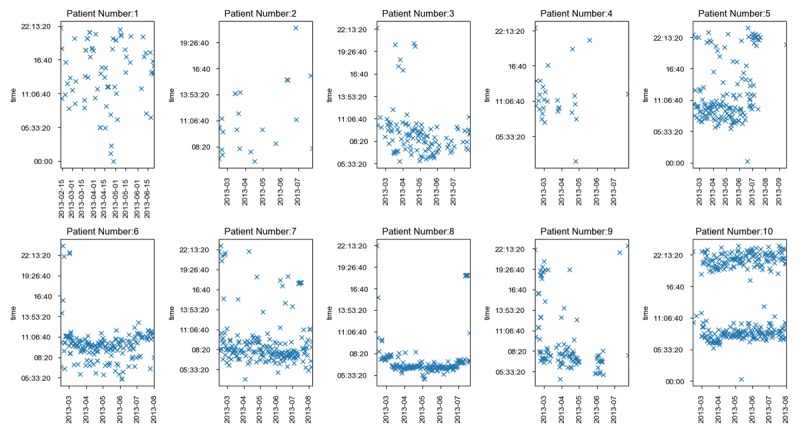
Time distribution of self-monitored blood glucose level collection by the patients over the clinical trial. The x-axis represents the date and the y-axis represents the time of the day that data has been collected. If the same date has two recorded times, that means the patient has collected their blood glucose twice in the same day.

**Figure 3:**
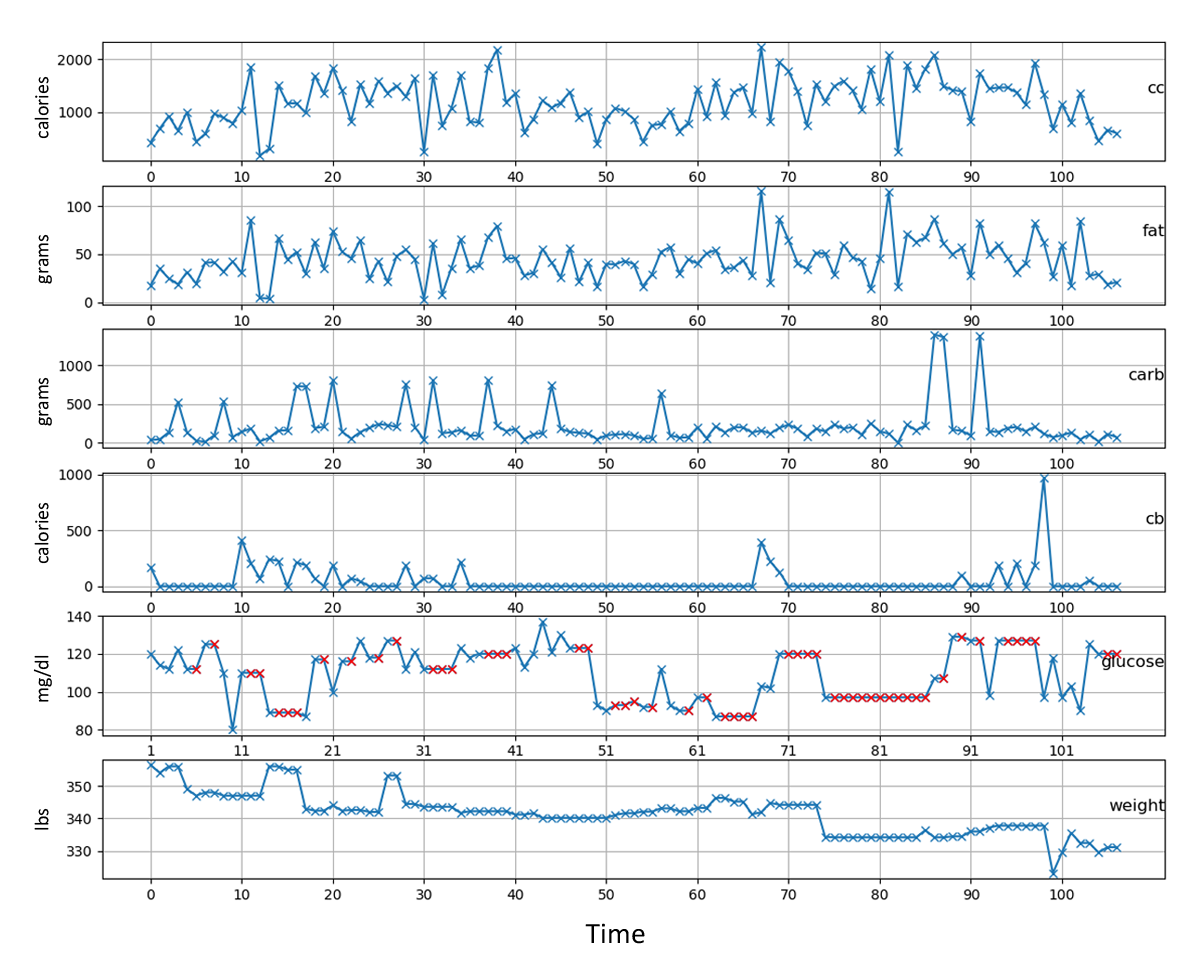
Patient 1 self-recorded data including calories consumed (cc), fat consumed (fat), carbohydratess consumed (carb), calories burned (cb), recorded blood glucose level for the day (glucose), and recorded weight for the day (weight), where blue markers represent original data points and red markers show the imputed data points.

Patients were not required to take glucose readings at a fixed time of day but were asked to be consistent in terms of collecting blood glucose readings every day. Figure 2 shows the distribution of each patient’s blood glucose recording time. For patients 3, 5, 6, 7, 8, and 9, the recorded times are generally between 8:00 am to 11:00 am. However, for patient 10, the recorded times are divided between 8:00 am and 10:00 am or 8:00 pm to 10:00 pm. For this patient, we considered the readings taken from 8:00 am to 10:00 am. For patients 1, 2, and 4, the number of recorded instances were fewer and scattered throughout the day. Figure 3 shows the self-monitored collected data for patient 1. While the patient has recorded their food intake for the day, they haven’t adhered to a daily exercise regimen, as can be seen from the calories burned (cb) subfigure.

**Table 1:** Descriptive statistics of glucose and weight levels for the patients in the study.

Patient #	Number of missing observations	Glucose level (mg/dL)			Weight (lbs)		
		Min	Max	Mean (SD)	Min	Max	Mean (SD)
1	118	80	137	110.99 (13.97)	323	356	341.60 (6.39)
2	161	108	171	136.15 (18.95)	225	241	230.42 (4.94)
3	73	275	87	151.00 (27.64)	144	148	145.62 (0.83)
4	154	137	192	155.97 (17.27)	170	183	178.38 (2.88)
5	48	73	287	132.79 (29.81)	304	312	30.06 (3.88)
6	29	85	306	196.62 (53.43)	178	191	184.39 (4.02)
7	16	72	200	119.95 (27.26)	273	248	260.40 (6.17)
8	26	111	168	138.10 (13.15)	150	155	152.97 (1.41)
9	102	74	1770	118.33 (39.98)	274	285	279.65 (3.15)
10	53	86	147	104.82 (10.80)	222	229	226.97 (3.17)

### Data Preprocessing

The study dataset is based on patient data entry with several complicating factors, including missing values, (possible) wrong entries, calculated features, and irregular sampling. Therefore, major preprocessing steps are needed to clean the data and make it compatible with the proposed deep learning model. The preprocessing steps considered for this study include handling of missing values, data scaling, and data splitting.

#### Handling Missing Values

The measurements in the database are sparse, irregularly sampled, and presented with missing data points. To handle missing values, assuming there is less chance of abrupt change in glucose level on the following day, the missing values of data were handled by replacing them with the last available data (last observation). Meanwhile, we noticed that many patients had a considerable number of missing values that could potentially affect the performance of the methods. To address this problem, we developed a transfer learning strategy to leverage the similarity between the information of patients to improve the predictions when dealing with data scarcity.

#### Data Scaling

**Figure 4:**
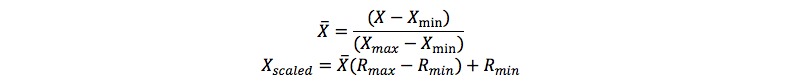
Equation for data scaling based on min-max normalization.

The range of values for each feature in the dataset varies extensively. Thus, the performance of the learning algorithm might be dominated by features with a wider range of values. The goal of this step was to scale the values of each feature within a predefined limit without losing the inherent information. For this purpose, we used data scaling based on min-max normalization [35] (Figure 4), where *X* denotes the original value of the feature of interest, *X_min_* denotes the minimum value of the feature, *X_max_* denotes maximum value of the feature, and *R* denotes the desired range of the scaled features, namely [–1,1].

#### Data Splitting

When making a dietary, physical activity, or medication plan for a patient, it is important to consider the time it takes for those changes to affect the patient. In order to provide enough time to observe patient behavior and test the model, we consider approximately 120 days of data for training, 30 days of data for validation, and 30 days of data for testing, where possible. For the patient with a smaller number of available records, we reduced the size of training, validation, and test sets proportionally. For example, for a patient with 41 days of entries, we considered 27 days of data for training, 7 days of data for validation, and 7 days of data for testing.

### Model Construction and Optimization: Long Short-Term Memory–Based Recurrent Neural Networks

**Figure 5:**
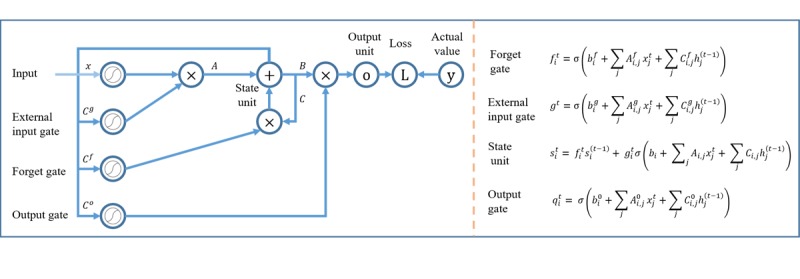
Block diagram of the LSTM neural network, where the left portion of the figure shows how an LSTM regulates the information flow using the three control gates and the right portion provides the mathematical equations for the key gates and units of the LSTM model. LSTM: long short-term memory.

We constructed a specialized RNN known as LSTM for predictive modeling of daily glucose levels using mobile health time series data. RNNs use the concept of parameter sharing across different layers and can effectively model data sequences of different lengths. However, classical RNNs suffer from the vanishing (often) or exploding (rarely) gradient information problem. Here, we consider an LSTM network that is explicitly designed to avoid the vanishing gradient problem by regulating the information flow using three distinct gates: forget gate, external input gate, and output gate. The forget gate (*f_i_^t^*) is a linear self-loop weight that decides which information to keep and which to drop. The external input gate (*g_i_^t^*) helps with deciding which new information to update in an LSTM memory unit/cell. The output gate controls the extent to which the values in the cell are to be used to compute the output activation. The state unit (*s_i_^t^*) is calculated based on the forget gate and external input gate. The output unit (*q_i_^t^*) then provides the necessary information to predict the output (*y’^t^*), which is the predicted glucose level for the next day (Figure 5).

### Knowledge Transfer Across Patients

**Figure 6:**
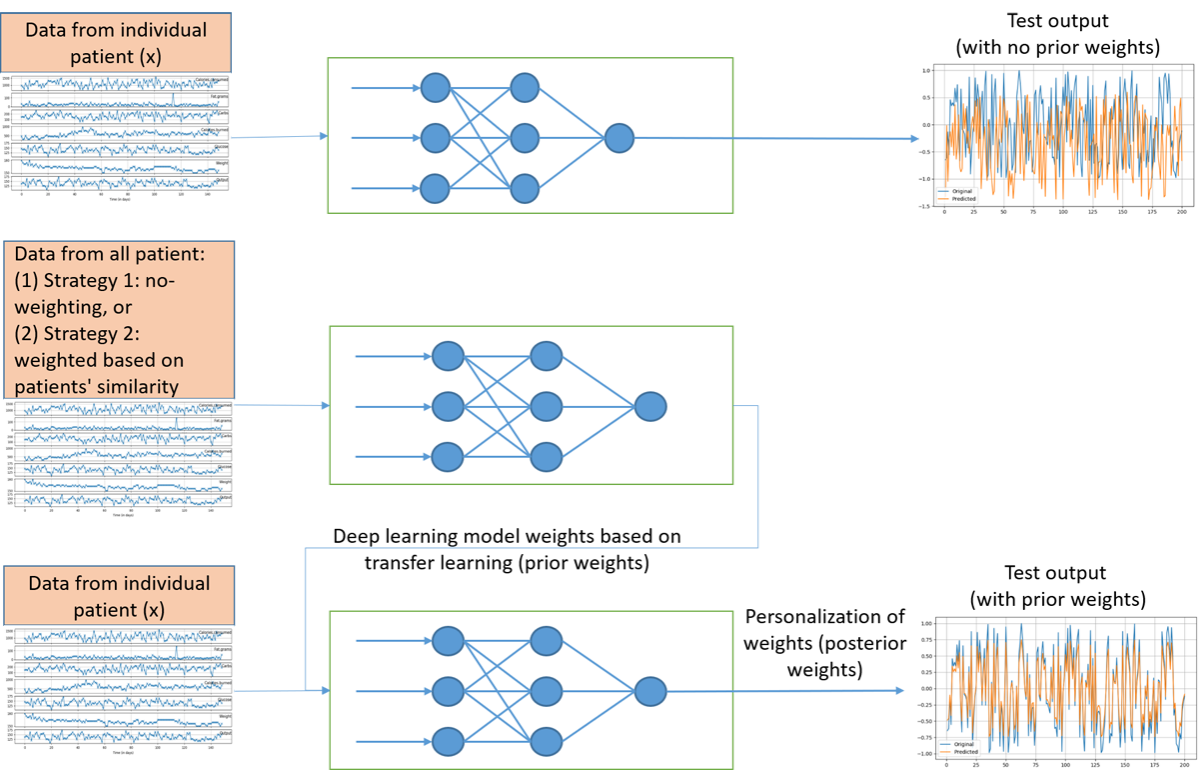
General scheme of the proposed transfer learning strategy. For patients with fewer observations, we pretrain a model with observations sampled from all the patients in the dataset (based on either no weighting or weighting strategy). The pretrained model is then fine-tuned with the data of the patient of interest.

It has been shown that transfer learning is useful in learning tasks when the data are scarce, contain many missing/imputed values, and/or suffer from complex patterns [36,37]. Here we developed two transfer learning strategies for coping with data scarcity and improving the predictions for individual patients. The first strategy used all the patient data (training data) to create the transfer learning dataset to pretrain a global LSTM model. The global model was then personalized for each patient based on their individual records. The second strategy used the similarity in glucose patterns between patients to create a transfer learning dataset for each patient. For that, a similarity matrix was created for each patient comparing their glucose patterns with all other patients using dynamic time warping (DTW) [38]. DTW is often used to compare two dynamic patterns and calculate their similarity by calculating the minimum distance between the two time series and aligning the significant patterns [39]. Next, it creates a transfer learning dataset for each patient by sampling records from other patients according to their similarity. It then uses the sampled data to train a deep learning model for the patient of interest, where the deep learning model weights of the trained model will be used as the prior. Finally, we personalized the deep learning model weights to the patient of interest using their own data. Figure 6 shows a visual representation of the proposed transfer learning strategies. In the results section, we compare the performance of the two transfer learning strategies to the no-transfer learning strategy.

### Model Selection and Parameter Tuning

The proposed LSTM model for prediction of glucose level has three hyperparameters to be optimized to achieve the best predictive performance. These three hyperparameters are dropout rate, number of neurons in LSTM layers, and number of neurons in the feed-forward neural network layers [40]. We considered lower bounds of (0.10, 5, 5) and upper bounds of (0.45, 60, 40) for the three hyperparameters, respectively. Optimizing the hyperparameters involved building, training, and validating many versions of the LSTM network based on various choices of the hyperparameters.

Considering an allowable unit change of 0.01 for the dropout rate parameter and 1 for the number of neurons in LSTM and feed-forward layers, we had to test a total of (35*55*35=67,375) combinations before finding the optimal hyperparameters, which was time-consuming. Thus, to identify the optimal value of the hyperparameters with a minimum number of trials and errors, we used an advanced design of experiments method based on Bayesian optimization [41]. The advanced design of experiment process began by generating a small sample of 15 experimental settings based on the three hyperparameters of the LSTM using a Latin hypercube design [42,43]. Next, for each of the initial set of 15 experimental settings, an LSTM network was built and tested over the validation dataset based on the root mean squared error (RMSE) of the actual versus predicted glucose level. Afterward, using the hyperparameters of the LSTM models as the input and the respective RMSE as the output, a surrogate model was fitted based on a Gaussian process. Then, the expected improvement criterion was used to identify the optimal point of the Gaussian process which represented the estimated optimal hyperparameter setting of the LSTM. The estimate of the optimal hyperparameter was used as the next hyperparameter setting to experiment [44]. This procedure continued until no improvement in the RMSE was observed. For the LSTM, expected improvement methods converged to the global optimum point after five iterations or 24 total evaluations (15 initial evaluations + 9 additional evaluations).

## Results

### Overview

We considered three variants of the proposed deep learning models for evaluation. The three variants were (1) the base LSTM-NN (without transfer learning), where the model was trained using only the respective patient’s data, (2) LSTM-NN-TF-ALL (first transfer learning strategy), where a general model was trained using all patients’ data and then personalized based on the target patient’s records, and (3) LSTM-NN-TF-DTW (second transfer learning strategy), where separate transfer learning datasets were created for training individualized models for each patient using similarity-based sampling from other patients’ records. We evaluated the performance of the deep learning models along with several baseline machine learning methods including an ANN [45], k-nearest neighbors (KNN) regression, ridge regression, kernel ridge regression with Gaussian kernel, and a moving average model. The validation dataset was used for tuning the hyperparameters of the comparing models, such as the optimal number of nearest neighbors in KNN (found at k=3), the sample size in the moving average (n=3), and the optimal value of the penalty term in the (kernel) ridge regression.

### Evaluation Criteria

Mean squared error and mean absolute error are commonly used to evaluate the performance of prediction models. However, these criteria do not consider the clinical impact of the prediction error and how it might affect medical decision making. Here, we considered two criteria which were related to the mean squared error and provided information about the clinical impact. The first criterion was the Clark Error Grid [46], which determined the acceptable error for the accuracy of blood glucose prediction in comparison with the actual observation. The second criterion, based on the prescription point of care [47], was the prediction accuracy within the range of ±10% of the actual value.

### Prediction Accuracy Based on the Clark Error Grid

The Clark Error Grid [46] is one of the most widely used tools to assess the clinical accuracy of blood glucose estimation. The Clark Error Grid is a plot with five major zone of attention (zone A, B, C, D, and E) for interpretation of the predicted glucose levels. Zone A represents those values within 20% of the reference value that generally lead to the appropriate treatment of patients. Zone B represents those values that are outside zone A, yet do not lead to inappropriate treatment of the patients. Prediction values falling in zone C lead to inappropriate treatment but without any dangerous consequences for the patient. Prediction values on zone D lead to failure in detecting hypoglycemia or hyperglycemia. Finally, prediction values in zone E lead to the inappropriate treatment of hyperglycemia instead of hypoglycemia and vice versa depending on the zone location.

Table 2 summarizes the percentage of prediction points falling in various zones of the Clark Error Grid for each of the comparing methods. As shown in the table, the proposed LSTM-NN-TF-DTW model has the highest percentage of predicted values in zone A (84.12%), followed by the kernel ridge regression (83.03%), and the moving average (82.01%). On the other hand, the moving average and kernel ridge regression have the lowest percentage of predicted values in zone C, D, and E, followed by LSTM and artificial neural network (ANN) models. Overall, ANN provides the lowest performance among all methods, which may be attributed to the large amount of data that it requires and the problem with vanishing gradient in RNN. Multimedia Appendix 1 (Figure C.1) complements Table 2 with visual illustrations of the Clark Error Grid for each of the comparing methods.

**Table 2:** Percentage of prediction points on the Clark Error Grid zones.

Clark Error Grid zone	LSTM-NN-TF-DTW^a^ (with transfer learning) (%)	LSTM-NN-TF-ALL^b^ (with transfer learning) (%)	LSTM-NN^c^ (without transfer learning) (%)	ANN^d^ (%)	KNN^e^ regression (%)	Ridge regression (%)	Kernel ridge regression (%)	Moving average (last 3 days) (%)
A	84.12	78.17	75.81	69.31	71.12	83.03	76.9	82.01
B	15.16	21.17	23.47	29.96	26.71	16.25	23.1	17.99
C	0	0	0	0	0	0	0	0
D	0.72	0.65	0.72	0.72	2.17	0.72	0	0
E	0	0	0	0	0	0	0	0

^a^LSTM-NN-TF-DTW: second transfer learning strategy.

^b^LSTM-NN-TF-ALL: first transfer learning strategy.

^c^LSTM-NN: without transfer learning.

^d^ANN: artificial neural network.

^e^KNN: k-nearest neighbors.

### Prediction Accuracy Based on the ±10% Range

Table 3 provides the predictive accuracy of the comparing methods based on the ±10% range of the actual values. As demonstrated in the table, LSTM neural networks generally outperform other methods by a margin of significance. Also, transfer learning strategies provide meaningful improvements to the LSTM network, with DTW transfer learning (weighted strategy) delivering better results. Meanwhile, there are a couple of exceptions, such as patients 5 and 8, where the moving average method makes better predictions. Further investigation of such cases reveals that those patients suffer from many (adjacent) missing values over a long period of time (see also Multimedia Appendix 1, Part D).

**Table 3:** Prediction accuracy of the proposed deep learning models along with other comparing methods based on the ±10% range of the actual glucose level value.

Patient #	LSTM-NN-TF-DTW^a^ (with transfer learning) (%)	LSTM-NN-TF-ALL^b^ (with transfer learning) (%)	LSTM-NN^c^ (without transfer learning) (%)	ANN^d^ (%)	KNN^e^ regression (%)	Ridge regression (%)	Kernel ridge regression (%)	Moving average (last 3 days) (%)
1	76.67	73.33	73.33	73.33	46.67	76.67	43.33	73.33
2	86.67	86.67	83.33	86.67	53.33	86.67	50.00	76.67
3	60.00	60.00	60.00	53.33	50.00	53.33	43.33	53.33
4	85.71	85.71	85.71	71.43	42.86	71.43	71.43	71.43
5	63.33	46.67	16.67	13.33	36.66	66.67	66.67	76.66
6	46.00	36.67	33.33	26.67	10.00	36.67	26.67	43.33
7	33.33	26.67	33.33	30.00	33.33	26.67	26.67	20.00
8	63.33	56.67	66.67	56.67	46.67	60.00	43.33	80.00
9	60.00	16.67	60.00	20.00	26.67	40.00	20.00	40.00
10	73.33	70.00	73.33	73.33	56.67	56.67	63.33	63.33

^a^LSTM-NN-TF-DTW: second transfer learning strategy.

^b^LSTM-NN-TF-ALL: first transfer learning strategy.

^c^LSTM-NN: without transfer learning.

^d^ANN: artificial neural network

^e^KNN: k-nearest neighbors.

## Discussion

### Principal Findings

The objective of this study was to dynamically forecast the next-day glucose levels in patients with T2DM based on their daily mobile health data including diet, physical activity, weight, and glucose levels from the day before. To achieve this objective, we developed an LSTM-based RNN that leverages these data and finds the pattern of glucose level change. We also developed two transfer learning strategies to deal with the issue of data scarcity and/or when a new patient starts using our model. The numerical results show the transfer learning model provided better prediction accuracy, especially in cases where there weren’t enough data available (and for patients with high variability). This provided the intuition for building an initial model that worked as a prior while collecting more data to personalize the predictions. Additionally, we used advanced design of experiments to optimize the hyperparameters of the proposed deep learning models with minimum effort. The proposed deep learning models performed well in comparison with the baseline models such as Kernel ridge regression and KNN. This pilot investigation has significant implications for future research studies in using real-world patient-generated lifestyle data to predict blood glucose changes to achieve optimal diabetes management.

The modeling of our study was closer to daily life in the real world involving dynamic data of physical activity, food intake, calories burned, body weight, and blood glucose generated by patients for 6 months. Previous studies have extensively focused on predicting glucose level for T1DM by engineering an artificial pancreas and simulating its insulin delivery to assist with the glycemic control of T1DM, and most predicting approaches are building upon physiological modeling [48]. Unlike T1DM, which is characterized as absolute inadequate insulin secretion of the body, the management of T2DM is largely determined by lifestyle [49-51]. Meanwhile, unlike the existing literature on forecasting glucose for T2DM based solely on food intake or glucose [52,53], our proposed model forecasts future glucose level more comprehensively by considering dietary habits, physical activity, weight, and previous glucose levels for patients and might provide a practical guide to T2DM management. We admit that blood glucose level can be affected instantly by an extreme lifestyle event (eg, a large amount of carbohydrate consumption or high levels of intensive physical activity without sufficient carbohydrate supplementation) [54,55]. However, our goal is to guide a patient through lifestyle changes (or choices) steadily, considering changing lifestyle choices takes time. Thus, the intention of this model was not to predict short-term blood glucose level variation throughout the day. Instead, it was designed to manage routine lifestyle in T2DM patients, and it is important to help patients understand how their lifestyle behaviors may change their blood glucose level in the very next day.

Glucose prediction was personalized in this model. Even though we have not counted all variations of each individual (demographic conditions, family history of diseases, etc), the prediction model does consider previous blood glucose level, which is the result of the interaction of lifestyle included in the model and other unexamined factors (eg, genes) [56]. In particular, the current glucose level may improve our understanding of glucose dynamics in patients with diabetes and serve as a crucial predicting factor for a future glucose level. For example, in T1DM patients, continuous glucose monitoring (CGM) is evidenced to predict future glucose level with high accuracy [49,57]. While CGM is generally not available and is not currently recommended for all T2DM patients [58], it is promising that CGM will be available with low-cost and noninvasive devices in the near future [59]. Using our model, as more data are recorded by the patient, the model will become more personalized to them, thus attaining higher accuracy in terms of predicting glucose levels, especially short-term glucose levels, throughout the day. Together with future advancements in characterizing biological traits, a more personalized and proactive diabetes management program will likely become practical. Our study has provided a promising piece of precision health, integrating dynamic lifestyle and daily glucose monitoring. Moreover, by using the prediction model to assimilate the massive amount of lifestyle data, health care providers can provide T2DM management guidance to patients without personally reviewing the data collected by mobile health technologies. This further makes precision health more feasible.

However, it should be noted that the prediction accuracy was low or modest for some participants. There are several possible explanations. First, glucose levels are multifactorial, and it might be hard to predict with limited input. Adding other patient features (eg, age, genetic profiling, demographic conditions, medication usage) [60,61] may increase the accuracy of prediction. Second, individual variability, such as genes and individual differences in glycemic response to lifestyle, further complicate the prediction. Studies are needed to explore models to mimic the interactions among predisposed traits (eg, genes), new input (eg, lifestyle), and the interactions between the two. Third, it could also be that blood glucose has a stronger dependency on short-term lifestyle choices than on long-term choices. For example, if a patient decides to consume a lot of carbohydrates or consume carbs at irregular times or work out irregularly, the glucose value would be unpredictable because there is no such information in the model. This further reinforces the importance of our study trying to predict future glucose levels and guide individual lifestyle choices. We would suggest that a more rigorous study design is needed to help identify the right model to predict future glucose levels. In particular, behaviors such as food consumption, physical activity, or medication use performed right before the predicting glucose level used to test prediction accuracy will need to be considered in the model. A model developed from a rigorous study design and data collection with high prediction accuracy may provide significant clinical implications to manage T2DM.

### Limitations

There are several limitations to this study. First, this study has a relatively small sample size, which may limit our study generalizability. Future studies with larger sample sizes are needed. Second, there are substantial variations in terms of accuracy when predicting blood glucose. Some underlying mechanisms, such as individual variations of age, gender, gut microbiota, and genetic traits [60-62], which are beyond the scope of this study, may have contributed to the variations. Future studies incorporating these factors are warranted. Third, given the nature of using secondary data from a previous trial, the data collection was not designed to predict future glucose levels. For example, the time of glucose level testing and diabetic medication use were not well documented and hard to include in the model. Last, several study participants had a large amount of missing data on glucose monitoring, and data were imputed using standard imputation methods widely used in the literature. Nevertheless, this is one of the first attempts of using digital monitored lifestyle data, weight, and previous glucose levels to predict future glucose levels in T2DM. It provides important information for future studies regarding data collection, model selection, and the implications of glucose prediction for individuals living with T2DM.

### Conclusion

In this work, we proposed a personalized dynamic forecasting model for glucose levels in T2DM patients based on LSTM-based RNN. We developed a transfer learning strategy based on weighted sampling from all patients to improve predictions, especially when dealing with data scarcity. We also used an advanced design of experiments based on Bayesian optimization and expectation maximization for efficient optimization of deep neural network hyperparameters with the minimum number of experiments. We tested our model using a longitudinal mobile health lifestyle dataset of 10 patients who provided self-monitoring data over 6 months on food intake (carbohydrates, fats, and calories), physical activity (exercise time and calories burned), weight, and previous glucose levels. Predicting future glucose levels is challenging as glucose level is determined by multiple factors. Future research with a more rigorous study design is warranted to help identify a model or models to predict future glucose levels.

## Appendix

Multimedia Appendix 1 Additional information on models, training procedure, and data analysis.

## Abbreviations

ANN: artificial neural network
CGM: continuous glucose monitoring
DTW: dynamic time warping
HbA_1c_: hemoglobin A1c
KNN: k-nearest neighbors
LSTM: long short-term memory
LSTM-NN: without transfer learning
LSTM-NN-TF-ALL: first transfer learning strategy
LSTM-NN-TF-DTW: second transfer learning strategy
RCT: randomized controlled trial
RMSE: root mean squared error
RNN: recurrent neural networks
T1DM: type 1 diabetes mellitus
T2DM: type 2 diabetes mellitus

## References

[1] Mougiakakou S, Prountzou A, Iliopoulou D, Nikita K, Vazeou A, Bartsocas C (2006). Neural network based glucose—insulin metabolism models for children with type 1 diabetes. Conf Proc IEEE Eng Med Biol Soc.

[2] Kovatchev B, Patek S, Dassau E, Doyle FJ, Magni L, De Nicolao G, Cobelli C (2009). Control to range for diabetes: functionality and modular architecture. J Diabetes Sci Technol.

[3] Olefsky JM (2001). Prospects for research in diabetes mellitus. JAMA.

[4] Narayan KM, Gregg EW, Fagot-Campagna A, Engelgau MM, Vinicor F (2000). Diabetes—a common, growing, serious, costly, and potentially preventable public health problem. Diabetes Res Clin Pract.

[5] van den Arend I, Stolk R, Krans H, Grobbee D, Schrijvers A (2000). Management of type 2 diabetes: a challenge for patient and physician. Patient Educ Couns.

[6] Nyenwe EA, Jerkins TW, Umpierrez GE, Kitabchi AE (2011). Management of type 2 diabetes: evolving strategies for the treatment of patients with type 2 diabetes. Metabolism.

[7] Blonde L (2005). Current challenges in diabetes management. Clin Cornerstone.

[8] Hunt CW (2015). Technology and diabetes self-management: an integrative review. World J Diabetes.

[9] Wang J, Cai C, Padhye N, Orlander P, Zare M (2018). A behavioral lifestyle intervention enhanced with multiple-behavior self-monitoring using mobile and connected tools for underserved individuals with type 2 diabetes and comorbid overweight or obesity: pilot comparative effectiveness trial. JMIR Mhealth Uhealth.

[10] Hanauer DA, Wentzell K, Laffel N, Laffel LM (2009). Computerized Automated Reminder Diabetes System (CARDS): e-mail and SMS cell phone text messaging reminders to support diabetes management. Diabetes Technol Ther.

[11] Padhye NS, Jing Wang (2015). Pattern of active and inactive sequences of diabetes self-monitoring in mobile phone and paper diary users. Conf Proc IEEE Eng Med Biol Soc.

[12] Nundy S, Dick JJ, Solomon MC, Peek ME (2013). Developing a behavioral model for mobile phone-based diabetes interventions. Patient Educ Couns.

[13] Arsand E, Tatara N, Østengen G, Hartvigsen G (2010). Mobile phone-based self-management tools for type 2 diabetes: the few touch application. J Diabetes Sci Technol.

[14] Wang J, Sereika SM, Chasens ER, Ewing LJ, Matthews JT, Burke LE (2012). Effect of adherence to self-monitoring of diet and physical activity on weight loss in a technology-supported behavioral intervention. Patient Prefer Adherence.

[15] Kebede MM, Zeeb H, Peters M, Heise TL, Pischke CR (2018). Effectiveness of digital interventions for improving glycemic control in persons with poorly controlled type 2 diabetes: a systematic review, meta-analysis, and meta-regression analysis. Diabetes Technol Ther.

[16] Faruque Labib Imran, Wiebe Natasha, Ehteshami-Afshar Arash, Liu Yuanchen, Dianati-Maleki Neda, Hemmelgarn Brenda R, Manns Braden J, Tonelli Marcello, Alberta Kidney Disease Network (2017). Effect of telemedicine on glycated hemoglobin in diabetes: a systematic review and meta-analysis of randomized trials. CMAJ.

[17] Clemens AH, Chang PH, Myers RW (1977). The development of Biostator, a Glucose Controlled Insulin Infusion System (GCIIS). Horm Metab Res.

[18] Aczon M, Ledbetter D, Ho L, Gunny A, Flynn A, Williams J (2017). Dynamic mortality risk predictions in pediatric critical care using recurrent neural networks.

[19] Plis K, Bunescu R, Marling C, Shubrook J, Schwartz F (2014). A machine learning approach to predicting blood glucose levels for diabetes management.

[20] Begg R, Kamruzzaman J, Sarker R (2001). Neural Networks in Healthcare: Potential and Challenges.

[21] Ma F, Chitta R, Zhou J, You Q, Sun T, Gao J (2017). Dipole: diagnosis prediction in healthcare via attention-based bidirectional recurrent neural networks.

[22] Che C, Xiao C, Liang J, Jin B, Zho J, Wang F (2017). An RNN architecture with dynamic temporal matching for personalized predictions of Parkinson’s disease.

[23] Hovorka R, Canonico V, Chassin LJ, Haueter U, Massi-Benedetti M, Orsini Federici M, Pieber TR, Schaller HC, Schaupp L, Vering T, Wilinska ME (2004). Nonlinear model predictive control of glucose concentration in subjects with type 1 diabetes. Physiol Meas.

[24] Georga E, Protopappas V, Fotiadis D, Funatsu K (2011). Glucose prediction in type 1 and type 2 diabetic patients using data driven techniques. Knowledge-Oriented Applications in Data Mining.

[25] Kok P (2004). Predicting blood glucose levels of diabetics using artificial neural networks [Thesis].

[26] Zitar R, Al-Jabali A (2005). Towards neural network model for insulin/glucose in diabetics-II.

[27] Tresp V, Briegel T, Moody J (1999). Neural-network models for the blood glucose metabolism of a diabetic. IEEE Trans Neural Netw.

[28] Li K, Daniels J, Herrero-viñas P, Liu C, Georgiou P (2018). Convolutional recurrent neural networks for blood glucose prediction.

[29] Mhaskar HN, Pereverzyev SV, van der Walt MD (2017). A deep learning approach to diabetic blood glucose prediction. Front Appl Math Stat.

[30] Quchani S, Tahami E, Ibraham F, Osman NA, Usman J, Kadri NA (2007). Comparison of MLP and Elman neural network for blood glucose level prediction in type 1 diabetics. 3rd Kuala Lumpur International Conference on Biomedical Engineering.

[31] Albers DJ, Levine M, Gluckman B, Ginsberg H, Hripcsak G, Mamykina L (2017). Personalized glucose forecasting for type 2 diabetes using data assimilation. PLoS Comput Biol.

[32] Hordern MD, Cooney LM, Beller EM, Prins JB, Marwick TH, Coombes JS (2008). Determinants of changes in blood glucose response to short-term exercise training in patients with type 2 diabetes. Clin Sci (Lond).

[33] Gibson BS, Colberg SR, Poirier P, Vancea DMM, Jones J, Marcus R (2013). Development and validation of a predictive model of acute glucose response to exercise in individuals with type 2 diabetes. Diabetol Metab Syndr.

[34] Aliberti A, Pupillo I, Terna S, Macii E, Di Cataldo S, Patti E, Acquaviva A (2019). A multi-patient data-driven approach to blood glucose prediction. IEEE Access.

[35] Rakthanmanon T, Campana B, Mueen A, Batista G, Westover B, Zhu Q, Zakaria J, Keogh E (2013). Addressing big data time series: Mining trillions of time series subsequences under dynamic time warping. ACM Transactions on Knowledge Discovery from Data (TKDD).

[36] Pan SJ, Yang Q (2010). A survey on transfer learning. IEEE Trans Knowl Data Eng.

[37] Yosinski J, Clune J, Bengio Y, Lipson H (2014). How transferable are features in deep neural networks?. http://papers.nips.cc/paper/5347-how-transferable-are-features-in-deep-neural-networks.pdf.

[38] Rakthanmanon T, Campana B, Mueen A, Batista G, Westover B, Zhu Q, Zakaria J, Keogh E (2013). Addressing big data time series. ACM Trans Knowl Discov Data.

[39] Salvador S, Chan P (2007). Toward accurate dynamic time warping in linear time and space. J Intel Data Anal.

[40] Srivastava N, Hinton G, Krizhevsky A, Sutskever I, Salakhutdinov R (2014). Dropout: a simple way to prevent neural networks from overfitting. J Mach Learn Res.

[41] Jones D, Schonlau M, Welch W (1998). Efficient global optimization of expensive black-box functions. J Global Optimiz.

[42] McKay MD, Beckman RJ, Conover WJ (1979). Comparison of three methods for selecting values of input variables in the analysis of output from a computer code. Technometrics.

[43] Pronzato L, Müller WG (2011). Design of computer experiments: space filling and beyond. Stat Comput.

[44] Rasmussen C, Nickisch H (2010). Gaussian processes for machine learning (GPML) toolbox. J Mach Learn Res.

[45] Chatterjee S, Dutta K, Xie H, Byun J, Pottathil A, Moore M (2013). Persuasive and pervasive sensing: a new frontier to monitor, track and assist older adults suffering from type-2 diabetes.

[46] Clarke WL, Cox D, Gonder-Frederick LA, Carter W, Pohl SL (1987). Evaluating clinical accuracy of systems for self-monitoring of blood glucose. Diabetes Care.

[47] Klonoff DC (2014). Point-of-care blood glucose meter accuracy in the hospital setting. Diabetes Spectr.

[48] McGrath T, Murphy KG, Jones NS (2018). Quantitative approaches to energy and glucose homeostasis: machine learning and modelling for precision understanding and prediction. JR Soc Interface.

[49] Taylor PJ, Thompson CH, Brinkworth GD (2018). Effectiveness and acceptability of continuous glucose monitoring for type 2 diabetes management: a narrative review. J Diabetes Investig.

[50] Sami W, Ansari T, Butt NS, Hamid MRA (2017). Effect of diet on type 2 diabetes mellitus: a review. Int J Health Sci (Qassim).

[51] Wing RR, Look AHEAD Research Group (2010). Long-term effects of a lifestyle intervention on weight and cardiovascular risk factors in individuals with type 2 diabetes mellitus: four-year results of the Look AHEAD trial. Arch Intern Med.

[52] Albers DJ, Levine M, Gluckman B, Ginsberg H, Hripcsak G, Mamykina L (2017). Personalized glucose forecasting for type 2 diabetes using data assimilation. PLoS Comput Biol.

[53] Martinsson J, Schliep A, Eliasson B, Meijner C, Persson S, Mogren O (2018). Automatic blood glucose prediction with confidence using recurrent neural networks.

[54] Esposito K, Ciotola M, Carleo D, Schisano B, Sardelli L, Di Tommaso D, Misso L, Saccomanno F, Ceriello A, Giugliano D (2008). Post-meal glucose peaks at home associate with carotid intima-media thickness in type 2 diabetes. J Clin Endocrinol Metab.

[55] Younk LM, Mikeladze M, Tate D, Davis SN (2011). Exercise-related hypoglycemia in diabetes mellitus. Expert Rev Endocrinol Metab.

[56] Lee PG, Halter JB (2017). The pathophysiology of hyperglycemia in older adults: clinical considerations. Diabetes Care.

[57] Frandes M, Timar B, Timar R, Lungeanu D (2017). Chaotic time series prediction for glucose dynamics in type 1 diabetes mellitus using regime-switching models. Sci Rep.

[58] Sakurai K, Kawai Y, Yamazaki M, Komatsu M (2018). Prediction of lowest nocturnal blood glucose level based on self-monitoring of blood glucose in Japanese patients with type 2 diabetes. J Diabetes Complications.

[59] Cappon G, Acciaroli G, Vettoretti M, Facchinetti A, Sparacino G (2017). Wearable continuous glucose monitoring sensors: a revolution in diabetes treatment. Electronics.

[60] Rich SS, Cefalu WT (2016). The impact of precision medicine in diabetes: a multidimensional perspective. Diabetes Care.

[61] Florez J (2016). Precision medicine in diabetes: is it time?. Diabetes Care.

[62] Utzschneider KM, Kratz M, Damman CJ, Hullar M (2016). Mechanisms linking the gut microbiome and glucose metabolism. J Clin Endocrinol Metab.

