# Supplementary material for: Development of a Deep Learning Model for Dynamic Forecasting of Blood Glucose Level for Type 2 Diabetes Mellitus: Secondary Analysis of a Randomized Controlled Trial
Source: JMIR Mhealth Uhealth. 2019 Nov 1;7(11):e14452. doi: 10.2196/14452 (PMC6858613; doi:10.2196/14452)
Supplement: Multimedia Appendix 1 [file mhealth_v7i11e14452_app1.pdf]

Appendices

Appendix A: Recurrent Neural Network

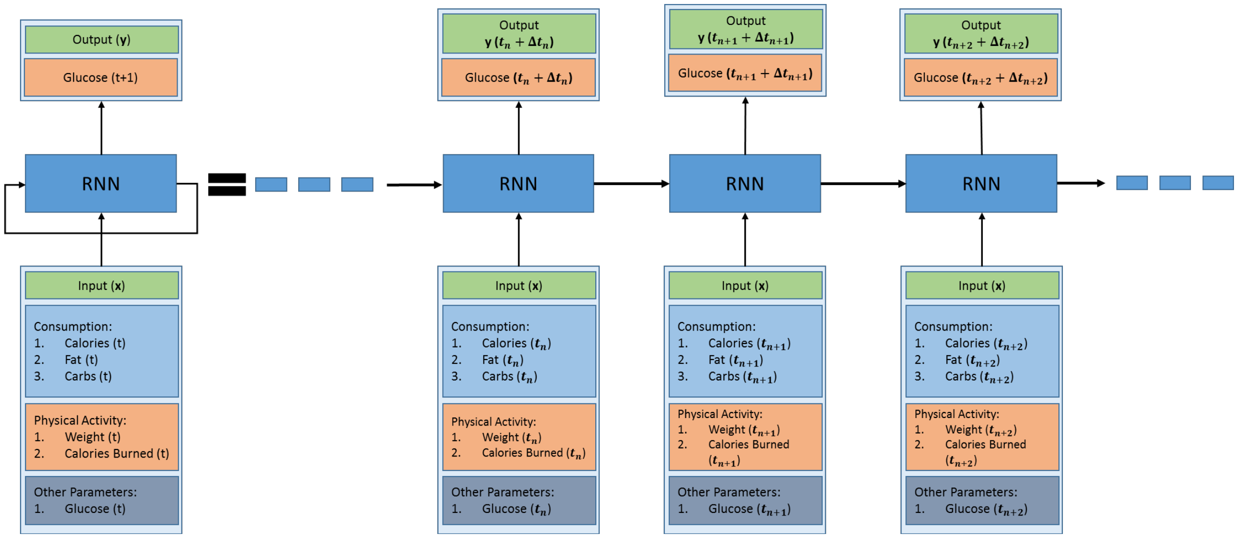

Figure A.1: A recurrent network

For analyzing the time series data, a recurrent neural network is used which specializes in processing a sequence of values. $X^1, X^2, X^3, \ldots \ldots \ldots \ldots, X^{t-1}, X^t$. Recurrent neural networks utilize the concept of parameter sharing across different layers of a model, thus making it easier to generalize the sequence length. Here, the notation, X^t, represents a vector which contains the sequence of data at any time limit, t. Let's consider the following form based on the problem at hand:

$$x^t = f\left(x^{(t-1)}, \theta_1, \theta_2, \ldots, , \theta_n\right)$$

Where, x is the state of the system and θ are the parameters affecting the state being considered. This is a recurrent system as the state x^t is dependent on the input parameter $x^{(t-1)}$, which is the state of the system at its previous time step. This can be unfolded by applying the definition for $t-1$. Such a recurrent process is shown in Figure A.1. The rolled model (left) shows that the model takes the three basic categories of data as input as well as the four self-monitoring variables (not shown on the figure). The unrolled model (right) shows that the model learns the patterns of time series at each time step, as well as shares the information with the next time step so that it can identify the patterns in an output model. The details of the proposed model's learning are discussed in later sections.

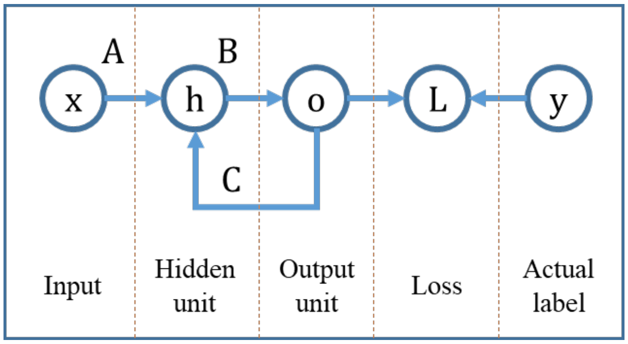

Figure A.2: The structure of the specialized recurrent network for predictive modeling of glucose level

Let b and c be bias vectors and A, B, and C be weight matrices of 'input to hidden', 'hidden to output' and 'hidden to hidden' connection respectively (see Figure A.2). For the following graph the updating equation will be (with the activation function being the hyperbolic tangent):

$$z^t = b + Cx^{t-1} + Ax^t$$
$$h^t = tanh(z^t)$$
$$\hat{y}^t = c + Bh^t$$

The total loss for a given sequence of x values paired with a sequence of y values would then be the sum of the losses over all the time steps. This can be written as a likelihood function as follows:

$$L(\langle X^1, X^2, \dots \dots, X^{t-1}, X^t\rangle, \langle y^1, y^2, \dots \dots, y^{t-1}, y^t\rangle) = -\sum_t log\ p\ (y^t | \langle X^1, X^2, \dots \dots, X^{t-1}, X^t\rangle)$$

The loss function is optimized using a gradient decent model.

Appendix B: Proposed Model and Training Procedure

Appendix B.1: Network Architecture and Hyperparameters

The final architecture used to predict the blood glucose level is shown in Figure B.1. Table B.1 shows the optimized hyperparameters used by the model to predict for each patient.

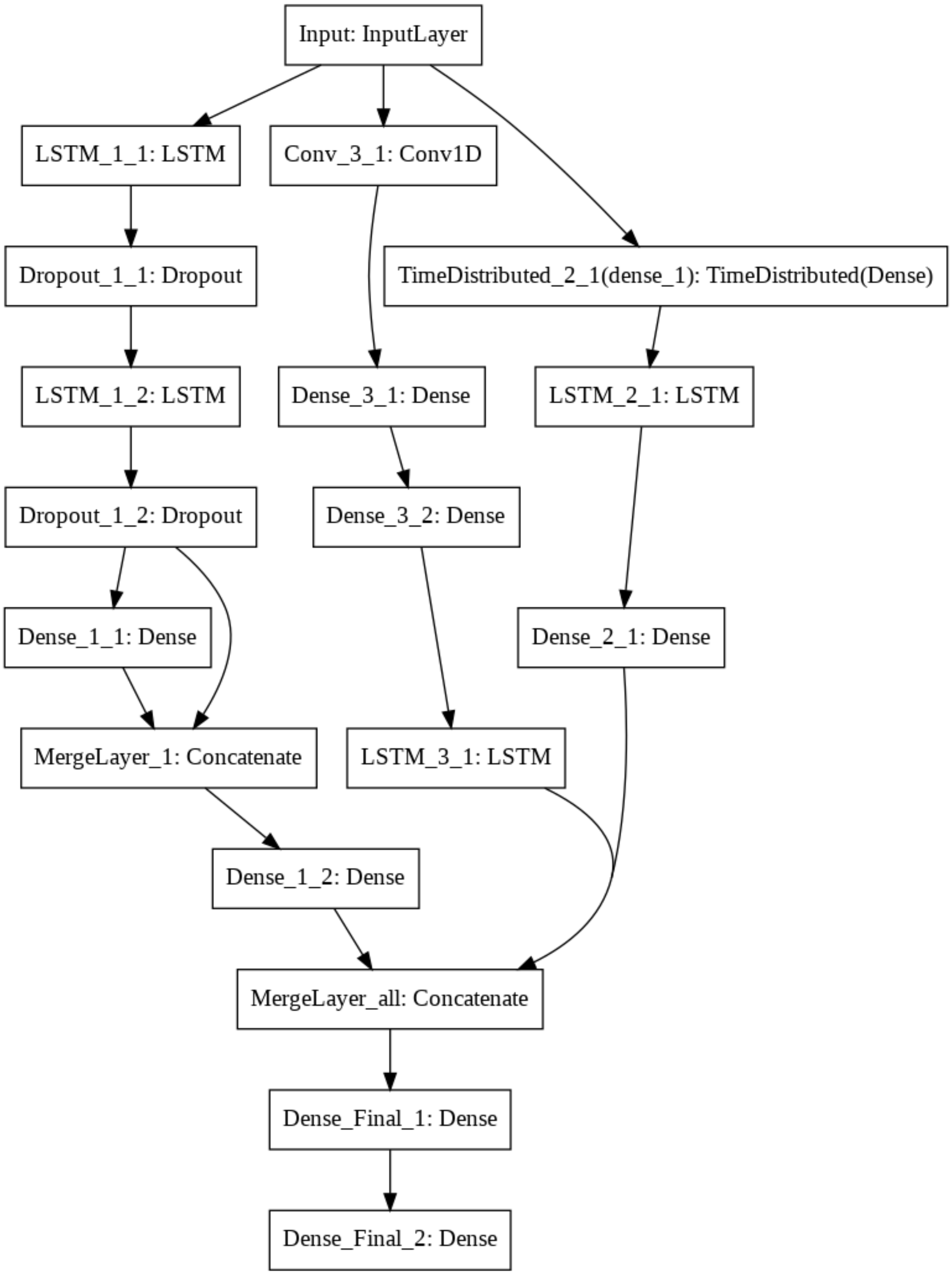

Figure B.1: The final architecture of the model used for predicting blood glucose level.

Table B.1. The optimized hyper-parameters attained using the advanced design of experiment to obtain the accuracy from the network for different patients.

Patient #	Dropout rate	neurons1	neurons2
**1**	0.23	33	31
2	0.43	5	16
3	0.14	8	31
4	0.44	9	28
5	0.14	8	31
6	0.39	24	10
7	0.43	5	16
8	0.14	8	31
9	0.35	52	36
10	0.44	9	28

Appendix B.2: Step by Step Training Procedure

Instead of a single generalized model for all 10 patients, a separate personalized LSTM model is considered for each of the 10 patients. The major steps of the training procedures resulted in the following setup steps:

1. A multi-branch multi-layer LSTM model (Check Figure B.1 for network architecture).
2. Data Pre-processing (as discussed in section *Data Preprocessing* of the main manuscript)
    a. The data is split in training, validation and test section-
        i. The model inputs at time, t are:
            1. Calories Consumed
            2. Fat Consumed
            3. Carbs Consumed
            4. Calories Burned
            5. Weight
            6. Glucose
        ii. The model output at time, $t + 1$ is the predicted blood glucose level.
    b. The training data is normalized within the range of (-1,1). Using the scale of the training data, the test data is transformed into a normalized form.
    c. Missing Blood Glucose levels were replaced as described in Handling missing value section.
3. The model only utilizes the past blood glucose level G_t i.e. it only uses the past observation instead of using a history of past sequences. This is done considering future blood glucose level is most likely to be affected by the last observed glucose level than a distance past observation.
4. The mean absolute error is minimized as an objective function using Adam backpropagation with a learning rate of 0.001 with a decay of 0.01.
5. The model hyper-parameters are optimized using advanced design of experiments methodology.

Appendix C: Clark Error Grid Analysis

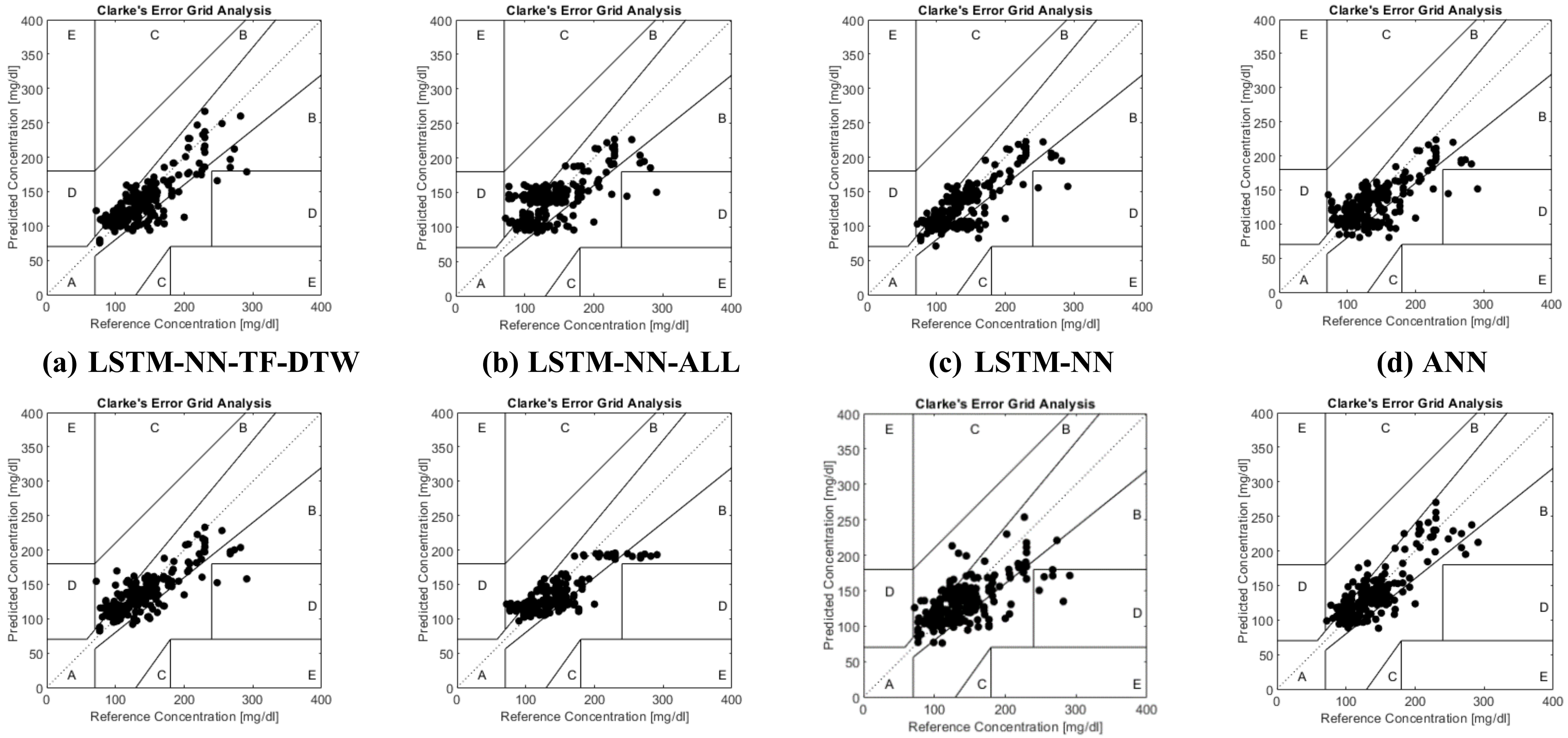

(a) LSTM-NN-TF-DTW

(b) LSTM-NN-ALL

(c) LSTM-NN

(d) ANN

(e) Ridge Regression

(f) Kernel Ridge Regression

(g) KNN Regression

(h) Moving Average (last 3 days)

Figure C.1: Error Grid Analysis of the predicted points. The plotted point shows the position of the predicted glucose level in different region of the error grid.

Appendix D: Prediction Plot

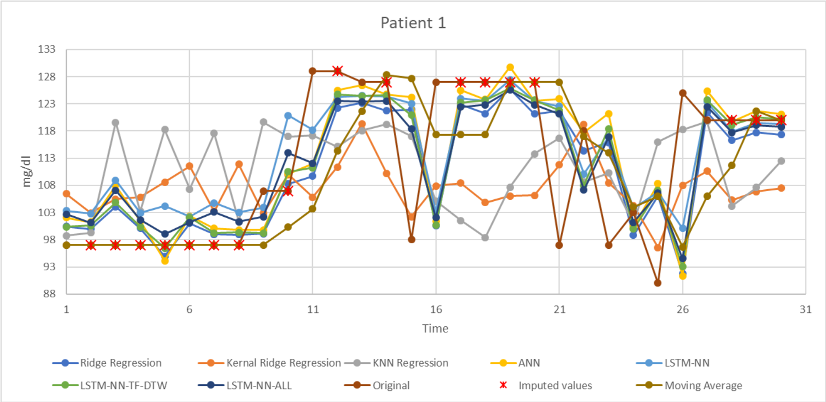

(a) Patient 1

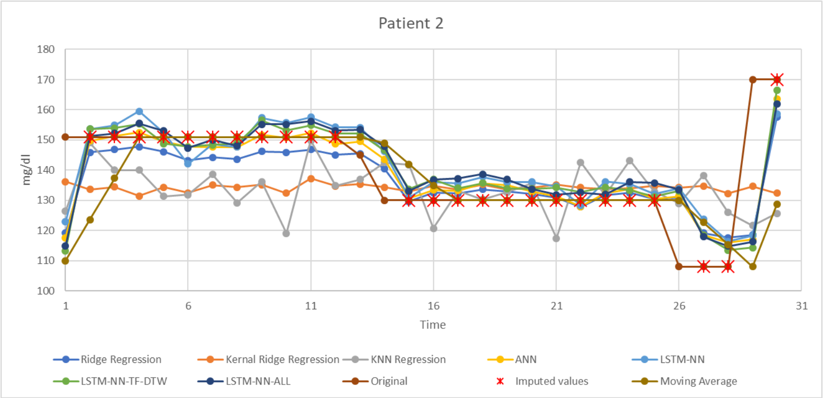

(b) Patient 2

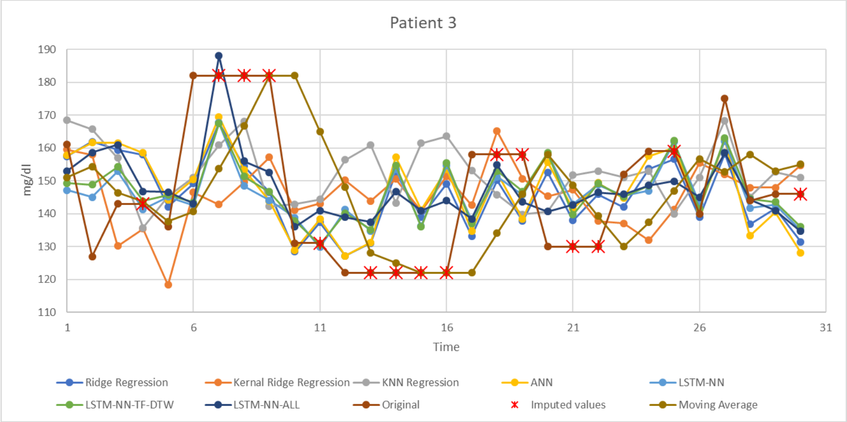

(c) Patient 3

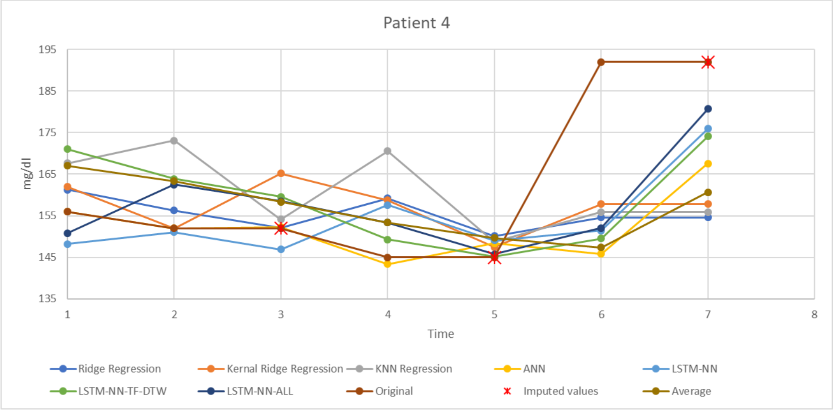

(d) Patient 4

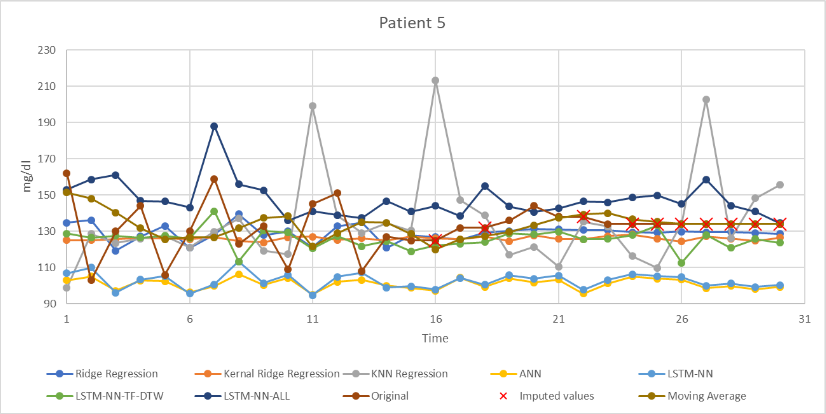

(e) Patient 5

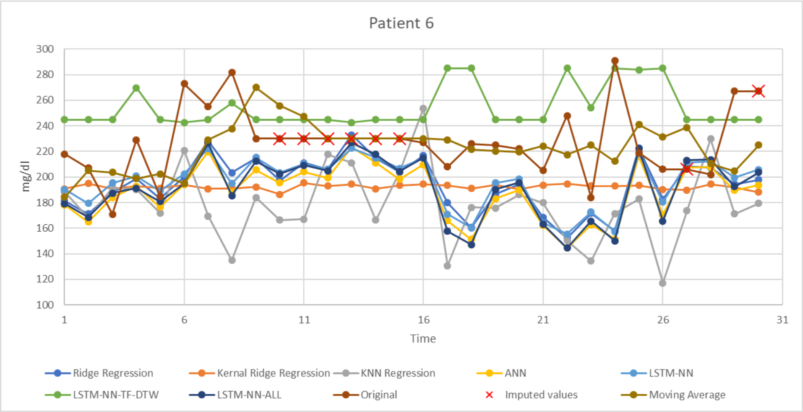

(f) Patient 6

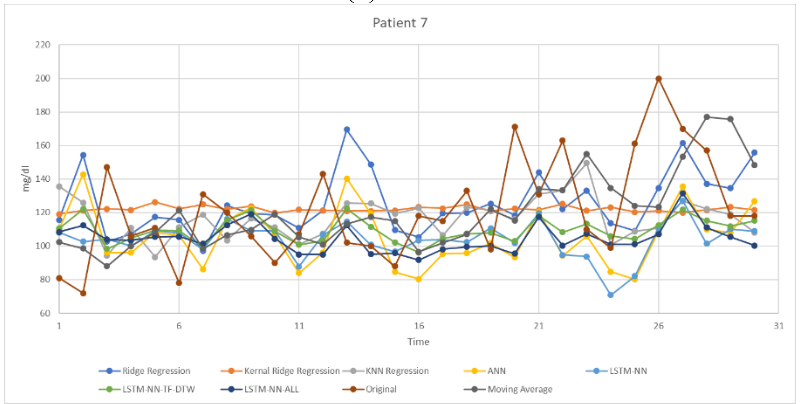

(g) Patient 7

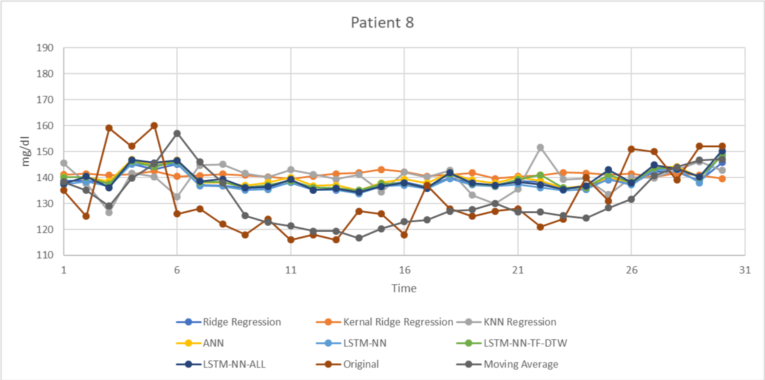

(h) Patient 8

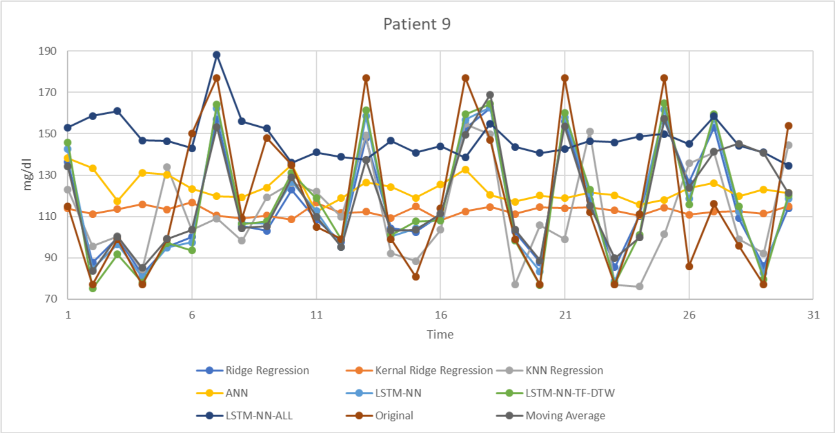

(i) Patient 9

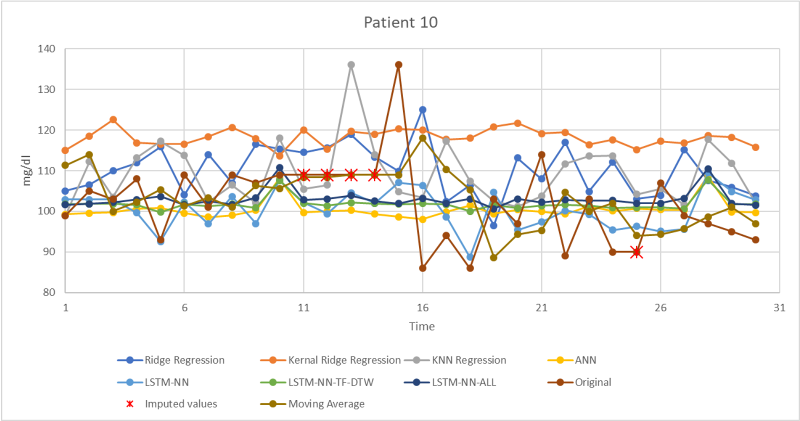

(j) Patient 10

Figure D.1: One day forward prediction results for the clinical trial of 10 patients. The solid brown line indicates the original data point and the other colors denote the predicted values. Imputed values are shown using red solid crosses.
